# Leukocyte- and Platelet-Derived Microvesicle Interactions following *In Vitro* and *In Vivo* Activation of Toll-Like Receptor 4 by Lipopolysaccharide

**DOI:** 10.1371/journal.pone.0025504

**Published:** 2011-09-26

**Authors:** Jing Xiong, Virginia M. Miller, Larry W. Hunter, Yunman Li, Muthuvel Jayachandran

**Affiliations:** 1 Department of Physiology, China Pharmaceutical University, Nanjing, Jiangsu, China; 2 Department of Surgery, Mayo Clinic, Rochester, Minnesota, United States of America; 3 Department of Physiology and Biomedical Engineering, Mayo Clinic, Rochester, Minnesota, United States of America; Tulane University, United States of America

## Abstract

**Background:**

Pro-coagulant membrane microvesicles (MV) derived from platelets and leukocytes are shed into the circulation following receptor-mediated activation, cell-cell interaction, and apoptosis. Platelets are sentinel markers of toll-like receptor 4 (TLR4) activation. Experiments were designed to evaluate the time course and mechanism of direct interactions between platelets and leukocytes following acute activation of TLR4 by bacterial lipopolysaccharide (LPS).

**Methodology/Principal Findings:**

Blood from age-matched male and female wild type (WT) and TLR4 gene deleted (dTLR4) mice was incubated with ultra-pure *E. coli* LPS (500 ng/ml) for up to one hour. At designated periods, leukocyte antigen positive platelets, platelet antigen positive leukocytes and cell-derived MV were quantified by flow cytometry. Numbers of platelet- or leukocyte-derived MV did not increase within one hour following *in vitro* exposure of blood to LPS. However, with LPS stimulation numbers of platelets staining positive for both platelet- and leukocyte-specific antigens increased in blood derived from WT but not dTLR4 mice. This effect was blocked by inhibition of TLR4 signaling mediated by My88 and TRIF. Seven days after a single intravenous injection of LPS (500 ng/mouse or 20 ng/gm body wt) to WT mice, none of the platelets stained for leukocyte antigen. However, granulocytes, monocytes and apoptotic bodies stained positive for platelet antigens.

**Conclusions/Significance:**

Within one hour of exposure to LPS, leukocytes exchange surface antigens with platelets through TLR4 activation. *In vivo*, leukocyte expression of platelet antigen is retained after a single exposure to LPS following turn over of the platelet pool. Acute expression of leukocyte antigen on platelets within one hour of exposure to LPS and the sustained expression of platelet antigen on leukocytes following a single acute exposure to LPS *in vivo* explains, in part, associations of platelets and leukocytes in response to bacterial infection and changes in thrombotic propensity of the blood.

## Introduction

Acute and chronic infection, especially that induced by Gram-negative bacteria is associated with increased risk of thrombosis and atherosclerotic disease [1], [2], [3], [4], [5]. Little is known about the underlying cellular mechanisms responsible for these risks. Lipopolysaccharide (LPS), a component of the cell wall of Gram-negative bacteria, is an antigen which initiates inflammation and innate immune responses by interacting with Toll-like receptor 4 (TLR4). TLR4 is expressed on the surface of cells, including leukocytes and platelets [6], [7], [8]. Under physiological conditions, platelets and leukocytes circulate in quiescent state and do not interact with each other. However, once activated under pathophysiological conditions such as those associated with infection, platelets change shape, secrete prothrombogenic inflammatory and cellular adhesion molecules from alpha- and dense-granules which cause the platelets to adhere to each other or to leukocytes and/or vascular endothelium [9], [10], [11], [12]. The physiological consequences of stimuli associated with infection, like LPS stimulation, are acute but can be sustained. For example, half-life of platelets was shortened and the activation state of newly formed platelets from bone marrow megakaryocytes increased within seven days following a single acute *intravenous* injection of LPS in mice [13], [14]. However, cellular events, specifically those occurring among blood elements, contributing to the shortened half-life and increased activation state of platelets remains to be clarified.

One mechanism offered to explain how infection contributes to the onset and progression of cardiovascular diseases is through increased production of proinflammatory cytokines [1], [3]. However, this explanation does not address how the production of inflammatory cytokines might proceed nor does it identity the cell types which are targets for the LPS stimulation. Platelets may represent one of the first blood borne elements to react to LPS stimulation as changes in platelet reactivity via TLR4 seems to occur prior to sustained changes in circulating levels of cytokines [14]. Alternatively, comparable activation of leukocyte as well as platelet result in formation of cell-derived microvesicles (MV) which may contribute to increased thrombogenic propensity of the blood, pro-inflammatory immune processes and thus cardiovascular risk [15], [16], [17], [18], [19], [20], [21], [22]. Clarifying the interactions of these blood elements (platelets and leukocytes) in the setting of TLR4 activation might provide insight into how infection initiates or facilitates progression of cardiovascular disease.

MV are cell membrane-derived vesicles ranging in size from 0.1 to 1 micron in diameter which are shed in response to cellular activation, cell-cell interaction and apoptosis [23], [24], [25], [26], [27]. These cell-derived vesicles are an interface of activation between cellular components of the blood with the vascular wall and between soluble components of the blood associated with immunity including response to infection [24], [28], [29]. For example, phosphatidylserine (PS) on the surface of MV provides catalytic sites for prothrombinase complex to generate thrombin needed for the conversion of fibrinogen to fibrin in formation of clots [25], [30], [31]. Furthermore, exposure of diluted blood to LPS *in vitro* increased production of platelet-derived as well as tissue factor positive MV within 3 to 6 hours [32], [33], [34]. While those experiments provide evidence that LPS modulates platelet activation, they do not provide any insight about the interactions of platelet with other blood elements within the earliest stages of activation especially at time points prior to the period when measurable changes in circulating cytokines are observed *in vivo* [14]. Therefore, the present study was designed to test the hypothesis that acute exposure to a sentinel dose of LPS would induce MV production and exchange of specific proteins/receptors between platelets and leukocytes via TLR4 activation. MV transport of biologically active cell contents including cell surface receptors among cells were identified using antibodies specific for cell antigens (i.e. CD41 or CD45, which is platelet- or leukocyte-specific antigens respectively) [24]; MV derived from platelets and/or leukocytes were distinguished by cell specific fluorescein conjugated antigen staining using calibrated flow cytometry.

## Materials and Methods

### Animals

Four to eight month old, male and female C57BL10SnJ mice (wild type, WT) and C57BL10ScN mice homozygous for deletion of TLR4 (dTLR4) were obtained from the Jackson Laboratory, Bar Harbor, Maine. These mice do not express the IL-12Rβ2 mutation that was originally described for this strain [35]. Mice of each sex and age were used randomly in each of the various protocols. Mice were housed in a temperature-controlled environment (22±2°C; 55±5% relative humidity), 12/12 light/dark cycle, and fed standard chow. Experiments were approved by the Institutional Animal Care and Use Committee, Mayo Clinic, Rochester, MN.

### Reagents

Ultra-pure E.coli lipopolysaccharide (LPS, 0111:B4 strain-TLR4 ligand, product number tlrl-pelps), pepinh-MyD (product number tlrl-pimyd) or pepinh-TRIF (product number tlrl-pitrif) inhibitory peptide (InvivoGen, San Diego, CA) were prepared as suggested by the supplier. Mouse thrombin and bovine serum albumin were purchased from Sigma Chemical Co., St. Louis, MO, USA. Cellular origin of antigens was determined using platelet (rat anti-mouse CD41 antibody) and total leukocyte (rat anti-mouse CD45 antibody) membrane specific fluorescein conjugated {Phycoerythrin (PE)- or fluorescein isothiocyanate (FITC)-} antibodies by flow cytometry. PE- or FITC-conjugated Annexin-V and matched isotype control antibodies were purchased from BD PharMingen International, San Diego, CA. All other reagents and solvents used in this study were of analytical/reagent grade.

### Experimental design and blood collection

Blood was collected from the retro-orbital sinus plexus (250–300 µL/mouse) of wild type and dTLR4 mice through siliconized capillary tubes coated with hirudin (thrombin inhibitor) and soybean trypsin inhibitor (STI, Factor Xa inhibitor) into 1.5 mL polypropylene tubes containing 20 µL of 100 µM hirudin and 1 mM STI [13]. For *in vitro* experiments, anticoagulated blood was aliquoted into pairs of tubes within 30 min after collection so that measurements from a vehicle-treated, control tube and LPS-treated tube could be analyzed from each mouse at each time point. Vehicle (saline) or LPS (500 ng/mL) was added to one of each paired aliquots of blood. At designated time points (5, 30, and 60 minutes) after addition of vehicle or LPS, 10 µL whole blood from each aliquot was diluted into 990 µL (1∶100) HEPES/HANKS' buffer (130 mM NaCl, 5.4 mM KCl, 1.3 mM CaCl_2_, 0.8 mM MgSO_4_, 0.44 mM Na_2_HPO_4_, 20 mM HEPES, pH 7.4) with 1 mg/ml albumin and 1 µM STI for staining of platelets positive for leukocyte antigen and leukocyte positive for platelet antigen. The remaining blood was used to prepare platelet free plasma (PFP) for MV analysis.

For *in vivo* experiments, male mice (8 month of age) received a single injection of LPS (500 ng/mouse or 20 ng/gm body weight) into the tail vein. Seven days after the injection, blood was collected as described above. For analysis of platelet antigen positive leukocyte sub-populations, flow cytometry was triggered with total leukocyte marker CD45-conjugated with allophycocyanin (CD45-APC). All CD45 positive events containing granulocytes, monocytes, lymphocytes and apoptotic bodies were separated by light forward and side scatter blot analysis and gated each sub-type of leukocytes based on their size. Each gate was verified using cell specific antibodies (CD11b for granulocytes, CD14 for monocytes and CD3 for T-lymphocytes and CD19 for B-lymphocytes. Differential leukocyte populations were then subjected to two-color analysis to differentiate leukocytes positive for phosphatidylserine and platelet antigen (annexin-V-FITC vs CD41-PE) from leukocytes negative for phosphatidylserine and platelet antigen. Percentages of positive events were calculated above the set threshold from isotype control antibody.

### Imaging mouse platelets

A blood sample from each mouse was divided into two 100 µl aliquots; one was diluted with 100 µl saline (control), the other with 100 µl saline containing LPS (500 ng/ml final concentration). Samples were incubated at 25°C. At time-points 0 (prior to LPS or vehicle), 30 and 60 min, a 20 µl aliquot was removed from each sample, diluted in 20 µl 2% paraformaldehyde and incubated for an additional 30 min. Each sample was then diluted in 250 µl PBS (0.1 µm pore membrane-filtered) and centrifuged at 45×g for 12 min. The supernate, which contained platelets, was removed, placed into a new vial and used for imaging analysis. An aliquot of each platelet suspension was diluted 1∶4 in PBS, and a 25 µl drop was placed on a glass slide, cover-slipped and sealed with glue. Platelets were then imaged in dark-field mode using a light microscope (Olympus BX41 with a 100× oil-immersion lens) coupled to a CytoViva illumination system (CytoViva, Inc., Auburn, AL). Scanning from a corner of each cover-slip, platelets in each field were counted until 100 were totaled. Platelets were categorized according to shape morphology (discoid, irregular, flattened, pseudopodia) or whether exhibiting membrane granules or in aggregates (each aggregate was counted as 1). The number of platelets in each category was expressed as percentage of total 100 platelets counted.

### Analysis of whole blood from *in vitro* experiments

**Figure 1:**
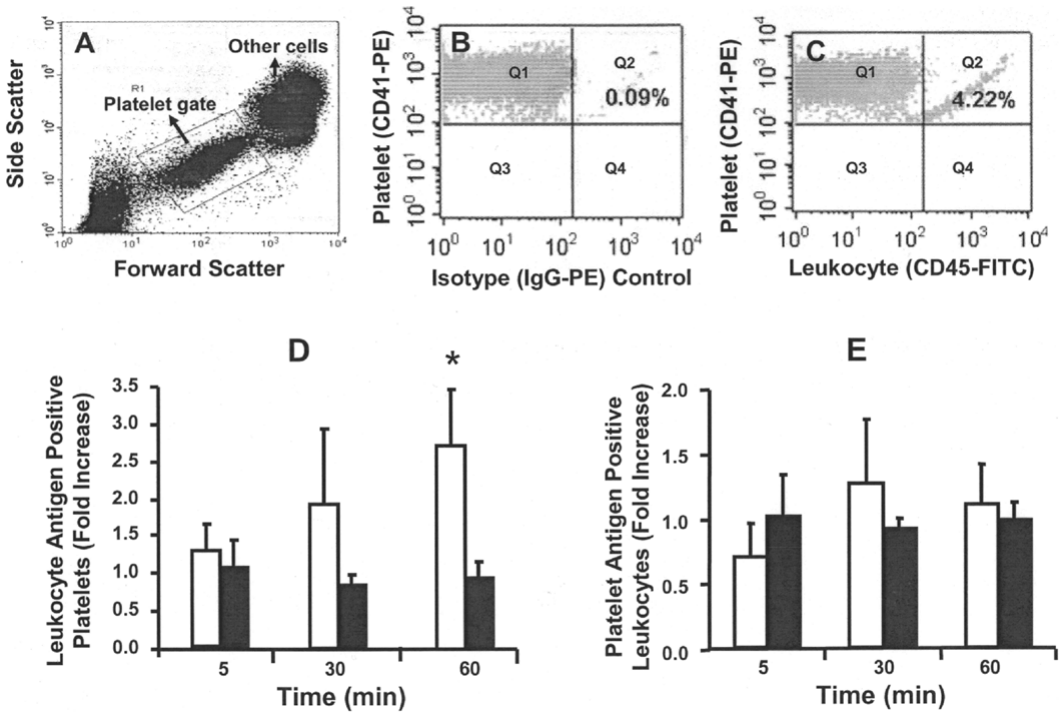
Identification of platelets and leukocytes positive for dual cell-specific antigens. A. Representative scatter plot of the gate used to identify platelets by size. B. Quadrants derived from platelet gate of blood stained with CD41-PE plus IGg-FITC (matched isotype control). C. Quadrants derived from platelet gate of blood stained with CD41-PE plus CD45-FITC (leukocyte-specific antibody). D & E. Cumulative data of platelets expressing leukocyte antigen (D) and leukocytes expressing platelet antigen (E) after incubation of blood with LPS (500 ng/mL) for up to 60 minutes. Data are derived from positive quadrants of platelet and leukocytes gates of scatter plots. WT mice (open bars); dTLR4 mice (closed bars). Data are expressed as mean ± SEM (n = 8/group) of fold increases from the vehicle-treated group at the same time point. *P<0.05 vs. saline-treated group at the same time point.

Diluted (1∶100) whole blood (100 µL) was incubated with PE-conjugated platelet- and leukocyte-specific antibodies (CD41 and CD45, respectively), or separately with annexin V-FITC (binding to phosphatidylserine) for 30 min, after which 1% paraformaldehyde (400 µL) was added. Matched fluorescein conjugated isotype control antibodies were used simultaneously staining to set the threshold and exclude nonspecific binding. Interactions between cell (platelets or leukocytes) and cell-derived MV and PS expression on both platelets and leukocytes were analyzed by flow cytometry (FACSCalibur™ and FACSCanto™, BD Biosciences, San Jose, CA). Platelets were identified by forward and side scatter and with fluorescein conjugated CD41 antibody until 20,000 events gated for each sample, respectively (Figure 1A).

### Microvesicles isolation and analysis

All buffers and antibodies were filtered twice through 0.2 µm membrane (Millipore) filters for MV analysis. Platelet free plasma (PFP) was prepared by double centrifugation at 3000×g for 15 min. PFP was diluted (15 µL PFP+85 µL HEPES/HANKS' buffer) and incubated with 4 µL FITC-conjugated annexin-V and PE-conjugated CD41 or CD45 antibodies for 30 min in dark, at which time 350 µL HANKS' balanced salts buffer (pH 7.4) with 2.5 mM CaCl_2_ was added. Matched isotype control antibodies were used to set threshold and exclude nonspecific binding. MV were quantified based on counts of calibration beads (TruCOUNT™ beads) added immediately to the samples prior to analysis by flow cytometry (FACSCanto™, BD Biosciences, San Jose, CA) as previously described [25], [26], [31]. In this study, MV were defined as events of <1 µm in diameter using size calibration beads and positive for annexin-V and platelet- and leukocyte- specific markers.

### Analysis of TLR4 intracellular signaling pathway

LPS stimulated TLR4 signaling was analyzed by introducing MyD88 or TRIF inhibitory peptides as described in other studies [36], [37]. Anticoagulated blood from WT mice was aliquoted into six tubes and treated with pepinh-control (vehicle), pepinh-MyD (100 µM, MyD88 inhibitory peptide which binds to MyD88 to block TLR4 stimulated MyD88 signaling) and/or pepinh-TRIF (100 µM which binds to TRIF to block TLR4 stimulated TRIF signaling) alone or in combination for 30 min after which either saline or LPS (500 ng/mL) was added for one hour. At this time, an aliquot was diluted for analysis by flow cytometry to measure cellular origin of MV and platelet positive leukocyte antigen and leukocyte positive platelet antigen.

### Data analyses

Data are presented as percentage or fold increase from the paired vehicle and LPS treated blood samples at each time point. All data are presented as mean ± SEM; n = number of animals used in each experiment. Statistical significance was evaluated by paired or unpaired two-tailed Student's *t*-test. Statistically significance was accepted at P<0.05.

## Results

### *In vitro* experiments

**Figure 2:**
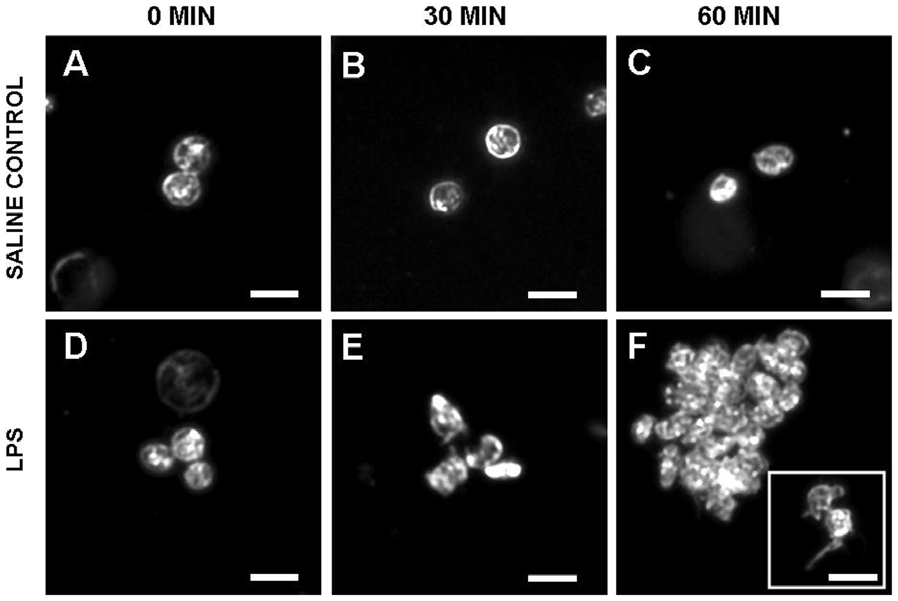
Representative dark-field micrographs of platelets from a WT mouse taken immediately before or after 30 and 60 min. incubation of the blood with 500 ng/ml LPS. Prior to LPS application, platelet aggregates (of more than 5 platelets) were absent, and most platelets were discoid shaped (Left panels). At 30 and 60 minutes incubation with saline, platelet morphology did not change (Top panels); whereas, large aggregates of irregularly-shaped platelets were observed in those incubated with LPS (bottom panels, inset shows platelets with pseudopodia). Each bar represents 5 µm. Similar results were observed with blood from two additional animals (n = 3).

Prior to addition of LPS, platelets from WT mice were discoid, formed few aggregates and did not have extended psuedopodia (Figure 2). Surface expression of PS on platelets or leukocytes was similar in WT and dTLR4 mice. Within each group of mice, expression of PS was significantly lower on leukocytes than on platelets (Table 1).

**Table 1:** Expression of PS aggregates and plasma MV before and after treatment with LPS (500 ng/ml for 60 minutes).

	Control		LPS (500 ng/ml; 60 min)	
	PS Expression (%)	Plasma MV (counts/µ L plasma)	PS Expression (%)	Plasma MV (counts/µ L plasma)
**WT Platelet**	4.16±1.54 (n = 7)	33.42±10.24 (n = 7)	2.51±1.01 (n = 4)	49.64±12.27 (n = 6)
**WT Leukocyte**	0.10±0.05 (n = 7)*	22.21±2.64 (n = 7)	0.08±0.05 (n = 4)*	27.14±2.81 (n = 4)
**dTLR4 Platelet**	3.50±0.87 (n = 7)	25.09±3.68 (n = 9)	2.83±0.64 (n = 5)	43.46±18.11 (n = 4)
**dTLR4 Leukocyte**	0.07±0.03 (n = 7)*	22.45±2.05 (n = 9)	0.04±0.008 (n = 5)*	31.17±2.43 (n = 4)

Data shown as mean ± SEM; PS, phosphatidylserine; MV, microvesicles; LPS, lipopolysaccharide; WT, wild type; dTLR4, gene deleted toll-like receptor 4.

*denote the significant difference in PS expression between platelets and leukocytes in the same genotype mice (p<0.05).

During the first hour of incubation of the blood with LPS, platelets from WT mice underwent a shape change, extended pseudopodia and formed aggregates (Figure 2). Within this first hour, neither surface expression of PS on either platelets or leukocytes or numbers of platelet- or leukocyte-derived MV changed significantly in WT mice or dTLR4 mice (Table 1). However, the number of platelets positive for leukocyte-specific antigen (defined by the platelet-gate on the flow cytometer, Figure 1) increased significantly in a time-dependent manner in blood of WT mice, but not dTLR4 mice (Figures 1). Using the total leukocyte specific antibody (CD45-APC) to define total leukocytes, numbers of leukocytes positive for platelet-specific antigen did not increase significantly in either WT or dTLR4 mice (Figure 1).

**Figure 3:**
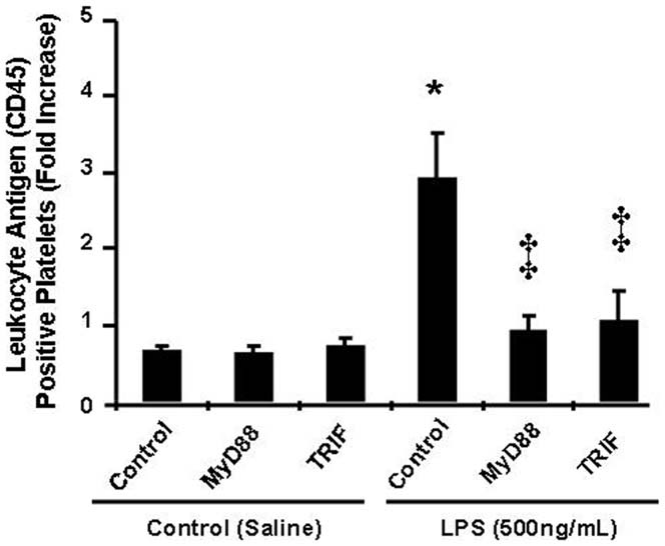
Quantification of platelets positive for leukocyte antigens using FASCalibur™ flow cytometry set to the platelet gate after incubation with inhibitory peptides. Cumulative changes in number of platelets positive for leukocyte antigens after incubation of whole blood with saline or LPS (500 ng/mL; for 60 min), in the absence or presence of control (100 µM) or inhibitory peptides MyD88 and TRIF (100 µM each). Data are expressed as mean ± SEM (n = 8/group). *P<0.05 vs. saline-treated group, ‡P<0.01 vs. control peptide-treated group incubated with LPS.

In blood from WT mice, the percentage of platelets positive for leukocyte antigen (CD45) was reduced significantly and to the same extent by MyD88 and TRIF (Figure 3). The combination of MyD88 with TRIF did not reduce the percentage of aggregates to a greater extent than either alone (from 3.83±0.48 to 1.38±0.08).

### *In vivo* experiments

**Figure 4:**
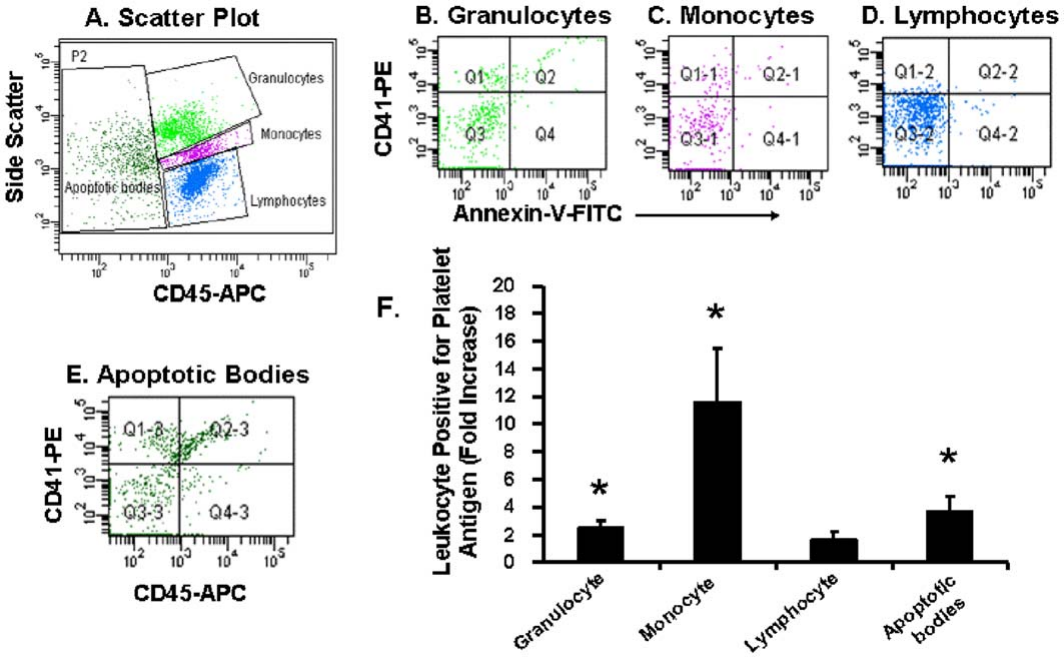
Sub-classes of leukocytes and apoptotic bodies expressing platelet antigen derived from blood of male mice seven days after a single intravenous injection of LPS (25 ng/gm body weight) or vehicle. A. Scatter plot of leukocyte triggered by CD45-APC. B. Quadrants derived from granulocyte gate. C. Quadrants derived from monocyte gate. D. Quadrants derived from lymphocyte gate. E. Quadrants derived from apoptotic bodies. F. Cumulative data expressed as mean ± SEM (n = 5) of fold increase from the vehicle-treated group of each sub-class of leukocytes positive for platelet antigen. Asterisks denote statistical significance (p<0.05) from baseline values, i.e. prior to the intravenous injections.

Seven days following a single intravenous injection of a sentinel dose of LPS (20 ng/gm body weight which is 500 ng/mouse), none of the platelets were positive for leukocyte antigen. On the contrary, compared to leukocytes obtained from animals treated with vehicle or a week before LPS injection in the same mouse, granulocytes and monocytes were significantly positive for platelet antigen seven days after the single LPS injection (Figure 4). Apoptotic bodies also stained positive for platelet antigen (Figure 4).

## Discussion

Understanding how infection alters cell-cell interactions and release of MV from specific blood borne elements may help to identify new targets for reducing cardiovascular/thrombotic risk with infection. This study demonstrates the acute, immediate interaction of platelets and leukocytes after incubation of whole blood with a sentinel dose of LPS through TLR4 signaling. Exchange of antigens and associations of specific cell-derived MV among cells is a mechanism for the transfers signaling molecules to specific cells. For example, MV derived from neutrophils induced platelet activation by binding to platelets GP1bα (Glycoprotein 1b α) via activated α_M_β_2_ on MV [38], while platelet-derived MV mediate leukocyte-leukocyte aggregation, activate leukocyte phagocytic properties and amplify leukocyte-mediated tissue injury in thrombotic and inflammatory disorders [39]. Results from the present study demonstrate that the number of platelets positive for leukocyte antigen increased within 60 min of exposure to LPS. This increase was not accompanied by increased expression of PS on cell or MV surface. Because expression of the leukocyte antigen on platelets was defined using the platelet size gate and platelet specific marker CD41 on the flow cytometry, larger leukocytes would be excluded. Therefore, these leukocyte antigens may represent a membrane exchange during platelet-leukocyte adhesion or adhesion of leukocyte-derived MV to the platelets. The half-life of whole platelet-leukocyte aggregates may be shorter and therefore, we did not determine whole platelet-leukocyte aggregates in this study.

With agonist binding, the TLR4 dimerizes and undergoes a conformational change required for the recruitment of signaling molecules [40], such as the adaptor molecules myeloid differentiation protein 88 (MyD88) and Toll-interleukin-1(IL-1) receptor domain containing adaptor inducing interferon-β (TRIF), which mediate MyD88 dependent or independent pathway respectively [41], [42]. LPS activates both MyD88 and TRIF pathways, which are important in the TLR4 mediated intracellular signaling [42]. The acute effects of LPS on platelet and leukocyte activation were most likely mediated through activation of TLR4 as platelet positive leukocyte antigen was not observed in blood from dTLR4 mice. Furthermore, leukocyte antigen expression on platelet was reduced by inhibition of MyD88- and TRIF-dependent pathways alone or in combination. These signaling pathways may be potential molecular targets to inhibit infection/inflammation induced interactions among formed elements in the blood.

A single TLR4 sentinel dose injection of LPS, such as used in this study, shortened platelet half-life and increased platelet production without increases in cytokine production within 3 hours of stimulation [14]. Although *in vivo* interaction of platelet- and leukocyte-aggregates with the vascular wall could stimulate or exacerbate proinflammatory immune responses [43], [44], the half-life of these cell aggregates is not known. A significant finding of the present *in vivo* study is that leukocytes sustain or retain platelet antigen seven days after an *in vivo* injection of LPS. This time point corresponds to turnover of the platelet pool and is consistent with observations that changes in platelet half-life and increases in platelet turnover are dependent on the concentration of agonist, i.e. LPS, activation [13], [14]. Quantification of dual-positive events is usually not considered in studies of cellular or MV quantification with LPS stimulation. Results from the present study, therefore, suggest that platelet-leukocyte antigen could be used as an additional biomarker of cellular activation for diagnostic or prognostic purposes in settings of sub-clinical or asymptomatic exposures to infective agents.

Monocytes showed the greatest expression of platelet antigen following LPS injection. Since monocytes are considered to be the primary leukocytic cell involved with development of atherosclerotic lesions [45], [46], [47], the present results provide insight into a mechanism linking low to moderate levels of infection to progression of cardiovascular disease.

To our knowledge, results of the present study represent the first to examine time-dependent changes in production of MV/exchange of cell-specific antigens in cells derived from whole blood incubated with LPS. In diluted blood, addition of LPS (500 ng or 1 µg/mL) increased the number of MV from platelets and those positive for tissue factor but only after four hours of incubation [34]. Because the blood was diluted, unlike the present study, cell-cell interactions were most likely attenuated and no evaluation was performed relative to interaction with leukocytes which could have indirectly affected platelet activation and production of cytokines.

Unexpectedly, PS expression on platelets (or leukocytes) did not increase significantly with acute exposure to LPS. PS is expressed in the inner leaflet of plasma membrane but rapidly inverts to outer surface following activating stimuli [48], [49], [50]. Exposure of PS to the outer membrane surface also occurs with release of MV [51]. Results of the present study are consistent with reports that production of MV is not accompanied by PS expression on cell of origin but might be restricted to that portion of the membrane undergoing MV blebbing [52].

### Conclusion

Within one hour of exposure of whole blood to a concentration of LPS that has threshold effect on cytokine production *in vivo*, platelets become positive for leukocyte antigen. Platelet-leukocyte interactions require TLR4 signaling as the dual antigen positivity of platelets was observed in blood derived from wild type but not dTLR4 mice. Furthermore, peptide inhibitors of TLR4 signaling molecules blocked the interaction. These events occur within 1 hour after the initial exposure to LPS. In addition, effects of LPS stimulation are sustained at least up to 7 days past the initial LPS exposure, as leukocytes express platelet antigen at this time point. Collectively, these results identify an acute and rapid signaling mechanism by which sentinel-grade acute infection through TLR4 alters blood hemostasis and sustained leukocyte activation which may contribute to progression of cardiovascular diseases.
